# Can an Accelerometer-Based Monitor be used to Accurately Assess Physical Activity in a Population of Survivors of Critical Illness?

**DOI:** 10.5539/gjhs.v4n3p98

**Published:** 2012-05-01

**Authors:** Lara Edbrooke, Noel Lythgo, Unna Goldsworthy, Linda Denehy

**Affiliations:** 1 Department of Physiotherapy, The University of Melbourne, Victoria, Australia; 2 School of Medical Sciences, RMIT University, Victoria, Australia

**Keywords:** intensive care, gait, motor activity, rehabilitation

## Abstract

**Purpose:**

To investigate the validity and reliability of the Activity Monitoring Pod (AMP331) to record gait parameters in healthy young adults (YA) and intensive care unit inpatients (ICU).

**Methods:**

YA (N=15) completed a series of over-ground walks. Another 15 YA completed a series of treadmill walks. The ICU group (N=20) completed a series of over-ground walks with repeat trials. Gait parameters were recorded simultaneously by the AMP 331 and the Vicon (YA) and the AMP 331, direct observation and a stopwatch (ICU).

**Results:**

For the YA over-ground, no significant differences were found between the measures recorded by the systems. For the YA treadmill, 43% of the measures differed (*P* < .05). For the ICU group, the AMP331 underestimated distance and speed by 3m and 25cm/s respectively. Reliability measures (ICU group) for distance (ICC 0.99, 95%CI 0.98 – 0.99) and step count (ICC 0.99, 95%CI 0.99 – 1.00) were excellent.

**Conclusions:**

The AMP 331 is a valid instrument for recording basic gait parameters for over-ground walking in healthy YA and ICU survivors.

## 1. Introduction

Physical inactivity is a major risk for chronic disease (Hu, Tuomilehto, & Silventoinen, 2004; Manson, Nathan, & Krolewski, 1992; Paffenbarger, Blair & Lee, 2001) and disability (World Health Organisation, 2002). Chronic disease management currently accounts for a large use of healthcare resources (Australian Institute of Health and Welfare, 2009) that place a significant burden on community services and family. Survivors of an intensive care unit (ICU) admission are reported to have significant functional and cognitive impairments up to five years after discharge (Herridge et al., 2011) and post intensive care syndrome (PICS) is a new term used to describe these chronic sequelae (Needham et al., 2012). Increased physical activity (PA) is therefore a goal of management in this patient population. A recent review of studies assessing free-living steps per day in special populations did not identify any studies performed in a population of ICU survivors (Tudor-Locke, Washington, & Hart, 2009). To date, we have found no reports measuring levels of PA using accelerometry in ICU survivors.

Measuring PA levels and adherence to exercise regimens in patients with chronic disease is of importance to researchers and clinicians given our rapidly ageing population. Clinicians require an accurate method of measuring PA levels to provide information regarding a patient’s baseline level of function and subsequently to assess adherence to home walking and exercise programs. Questionnaires and activity logs are subjective, limited by recall bias (Macko et al., 2002) and have been found to be insensitive to change in people with low to moderate activity levels (Wareham & Rennie, 1998). Such methods are still valuable in representing the patient’s perspective on whether existing activities have become easier for them (Bestall, Paul, & Garrod, 1999; Lareau, Meek, & Roos, 1998). Pedometers have been used extensively in previous research to measure PA levels. However, whilst more accurate than activity logs (Shephard, 2003) they have been found to be inaccurate, underestimating step counts at slower walking speeds, particularly in chronic disease populations (Cyarto, Myers, & Tudor-Locke, 2004; Foster RC et al., 2005). Accelerometers may offer a more sensitive and accurate method for use in less active populations (Pitta et al., 2005).

The Activity Monitoring Pod (AMP) 331 (Dynastream Innovations Inc., Cochrane, AB, Canada) accelerometer measurement accuracy during over-ground walking has previously been reported as 94% for distance and 99% for step count in a healthy population (Gildenhuys, MacDonald, Fyfe, & Stergiou, 2004). However, comparison with a criterion measure such as the Vicon Motion measurement system (Oxford, UK) has not been undertaken.

Further research into the validity and reliability of the AMP331 in measuring spatio-temporal gait parameters is required. The Vicon has been found to be valid in measuring distance compared to direct measurement (Ehara, Fujimoto, Tanaka, & Yamamoto, 1995) and as such was considered the criterion standard. To our knowledge, no previous studies have compared the AMP331 to the Vicon in any population. Previous studies have examined the validity and reliability of other brands of accelerometers during over-ground walking (Kuo, Culhane, Thomason, Tirosh & Baker, 2009; Le Masurier, Lee, & Tudor-Locke, 2004; Macko et al., 2002; Pitta et al., 2005; Ryan, Grant, Tigbe, & Granat, 2006) and treadmill walking (Le Masurier et al., 2004; Le Masurier & Tudor-Locke, 2003; Leenders, Nelson, & Sherman, 2003; Nichols, Morgan, Chabot, Sallis, & Calfas, 2000; Ryan et al., 2006; Welk, Almeida, & Morss, 2003; Welk, Schaben, & Morrow, 2004).

Limited assessment of the AMP331 at slower walking speeds has been reported. This is important as patients with chronic disease in early rehabilitation may walk slowly. The AMP331 has been used to record gait parameters in a group with type 2 diabetes following a rehabilitation program (Johnson, Tudor-Locke, McCargar, & Bell, 2005). Walking behaviour over a six day period in an elderly population with self-reported functional limitations has been studied using the AMP331 (Puthoff, Janz, & Nielsen, 2008).

This investigation had three primary aims. Firstly, to investigate the accuracy of the AMP331 accelerometer against the Vicon in measuring spatio-temporal gait parameters in over-ground walking at self-selected speed and during treadmill walking at a range of speeds. Secondly, to investigate the accuracy and reliability of the AMP331 to record spatio-temporal gait parameters in over-ground walking in a debilitated group of adults. Finally, to examine the test re-test reliability of the AMP331 accelerometer.

## 2. Methods

The predicted sample size based upon alpha = 0.05, beta = 0.9 and correlation of 0.75 (good to excellent reliability) is 12 subjects for each study (Portney & Watkins, 1993).

Fifteen healthy young adults (YA) (Mean ± SD; Age: 24.1 ± 9.1 yrs; Height: 172.3 ± 8.6 cm; Mass: 69.0 ± 14.2 kg) participated in the first study with another fifteen healthy YA participating in the second study (Age: 22 ± 7.8 yrs; Height: 173.3 ± 6.6 cm; Mass: 72.1 ± 12.7 kg). Participants were recruited from the University of Melbourne and were screened for recent musculoskeletal injury and neuromuscular dysfunction. Screening did not result in any young adult being excluded from the study. Ethical approval was obtained from the Human Research Ethics Committee of the University of Melbourne. Written informed consent was provided by each participant.

For the third study, twenty participants (Age: 62.1 ± 14.1 yrs; Height: 169 ± 9 cm; Mass: 81.1 ± 14.9 kg, Acute Physiological Chronic Health Evaluation II: 17 ± 6, ICU length of stay (LOS): 7 ± 4 days, acute hospital LOS: 37 ± 45 days) were sourced from a concurrent randomized controlled trial (RCT). Diagnoses (n) for this group were: post surgical (9), acute pulmonary oedema (2), septic shock (2), respiratory failure (2), pneumonia (2), multi-trauma (2), cardiac arrest (1). Eighty-five percent (17/20) of ICU AMP331 participants were classified as having chronic disease. Chronic disease was defined as having a part history of: respiratory, cardiac, liver or renal disease, diabetes, osteoarthritis, rheumatoid arthritis or ankylosing spondylitis. All participants were eligible to participate once they were able to walk a minimum of 5 metres without physical assistance. Participants were able to perform the trial protocol using a gait aid if required (N=15:no gait aid; N=4:single point stick; N=1:three-wheeled frame). Approval for this research was gained from the Austin Health Human Research and Ethics Committee (project number H2006/02424) Reporting of this research is in accordance with the STROBE statement for cross-sectional studies (www.strobe-statement.org).

### 2.1 Equipment

#### 2.1.1 AMP 331

The AMP331 records distance (m), speed (m/s), time (s), step count, step length (m) and cadence (steps/min). It classifies PA into three levels: inactive (no steps taken for ≥ 20 seconds), active (activities where < 20 steps are recorded i.e. housework) and locomotion (where ≥ 20 consecutive steps are recorded).

Data is downloaded to a Microsoft Excel spreadsheet. The AMP331 accelerometer was chosen for this study and the RCT for the following reasons: application ease, cost, ability to record for a 7-10 day period, ability to directly download data. In addition, findings from previous research show that ankle mounted devices, rather than hip mounted, have better accuracy for measuring step count at slow walking speeds (Karabulut, Crouter, & Bassett, 2005).

#### 2.1.2 Vicon Motion Measurement System

The 3D position of four passive spherical markers (diameter: 14mm) were recorded by an 8-camera Vicon sampling at 120 Hz. A 5000 mm (L) × 3600 mm (W) × 1800 mm (H) capture volume was calibrated for the over-ground walking task whereas a 3000 mm (L) × 2000 mm (W) × 2000 mm (H) capture volume was calibrated for the treadmill task. Mean spatial errors (i.e. distance parameters) have been found to fall around 2.3 mm (SD 1.2 mm) compared to direct measurement (Ehara et al., 1995) with temporal accuracy ranging from 0.02 to 0.002 seconds. Other work has reported spatial errors ranging between 0.8 and 1.3 mm (Windolf, Gotzen, & Morlock, 2008).

### 2.2 Protocol

Participant’s gender, date of birth, height and weight were recorded and programmed into the AMP331. In the first study, participants completed a series of over-ground walks at self-selected speed around a 14.76 m circuit (straight-length: 3.2 m; curve- length: 4.2 m) housed in a research laboratory. This track was the maximum volume captured for the Vicon. Initially, participants sat whilst the AMP331 was attached to the left ankle. It was held in place with a fabric pouch fastened by a Velcro strap. Four spherical passive reflective markers were then placed on the heel and toe regions with double-sided tape. Participants were instructed to walk at self-selected speed. They completed a practice lap followed by 3 trials that involved 2 (28.96m), 4 (57.91m) and 6 (86.87m) laps. These distances are representative of typical distances walked by healthy elderly (Bohannon, 1997). Participants began from a standing position (feet side-by-side) and finished at the same point. Upon completion of each trial, the AMP331 was removed and data extracted.

The same AMP331 and Vicon procedures were employed in the second study. Participants completed a series of walks on a Bodyworx JX325 motorized treadmill (GPI, Melbourne, Australia) set at speeds of 2.4, 3.4, 4.4 and 6 km·h^-1^ with an additional walk at 4.4 km·h^-1^ on a 5° incline. For each speed, the participants initially straddled the treadmill belt whilst the AMP331 was attached. The participant then walked on the treadmill for 3 minutes whereupon they straddled the treadmill belt whilst the AMP331 was removed for data download. Prior to the study, the treadmill speed and inclination were validated against the Vicon by recording the spatial position of a reflective marker placed on the treadmill belt. On average, the treadmill control gauge was found to overestimate the speed by 0.15 km·h^-1^ (SD = 0.07 km·h^-1^) and treadmill inclines of 0° and 5° were found to be 1.7° and 3.83° respectively.

In study three, participants walked at self-selected speed (AMP331 attached) along a hospital straight-line clearway for distances of 5, 10, 25 and 50m with each walk repeated twice. As participants were acute hospital inpatients they were unable to attend the research laboratory where the Vicon was located. Instead, control methods for the ICU group included timing of walks with a stopwatch (Digi Sport Instruments DT1, Pesotec Ltd, Hong Kong), measured distance and step counting by two observers. Participants were provided with a chair and instructed to sit at the end of each walk, whilst data were downloaded. Participants were instructed to notify researchers of any of the following new onset symptoms: chest pain, dizziness, unreasonable dyspnoea, light-headedness, extreme muscle fatigue or any other clinically warranted symptom. Participants were allowed to rest as required between walks.

### 2.3 Data Management and Analyses

In the first two studies, basic spatiotemporal gait parameters were simultaneously recorded by the AMP331 and the Vicon. These were distance walked, step count, speed, step length, cadence and time. These data were extracted directly from the AMP331. The foot marker 3D spatial coordinate data recorded by the Vicon were used to calculate the gait parameters. Visual inspection of a processed trial allowed the extraction of step count and trial time. Trial time was taken from gait initiation to termination. Cadence was derived from these measures. Distance for the over-ground condition was track length, whereas for the treadmill condition it was derived from adjusted treadmill speed (Table 2) and trial time. Step length was derived from distance and step count. Speed was derived from distance and time. In study three, step count was measured by direct observation and time with a stopwatch.

**Table 1:** AMP 331 and Vicon gait measures recorded during over-ground walking in healthy young adults

Parameter	2 Laps			4 Laps			6 Laps		

	AMP	Vicon	Mean difference	AMP	Vicon	Mean difference	AMP	Vicon	Mean difference
**Distance (m)**	27.93 ± 2.28	28.96	-1.02 (-2.29 to 0.24)	56.27 ± 3.56	57.91	-1.65 (-3.61 to 0.32)	84.20 ± 9.53	86.87	-1.34 (-1.30 to 3.98)
**Step count (steps)**	22.3 ± 1.80	22.60 ± 1.70	-0.33 (-0.68 to 0.01)	44.50 ± 4.10	44.70 ± 4.40	-0.20 (-0.57 to 0.17)	64.90 ± 5.40	65.10 ± 5.20	-0.20 (-0.51 to 0.11)
**Speed (cm·s^-1^)**	116.2 ± 16.60	116.0 ± 19.20	0.20(-5.50 to 5.80)	119.10 ± 19.40†	118.90 ± 21.80	0.20 (-5.00 to 5.30)	122.50 ± 20.90	123.40 ± 21.30	-1.00 (-9.50 to 7.80)
**Step length (m)**	63.20 ± 8.20	64.40 ± 5.20	-0.01 (-0.04 to 0.02)	63.50 ± 6.50	65.30 ± 6.50	-0.02 (-0.04 to 0.01)	65.20 ± 8.80	67.10 ± 5.40	-0.02 (-0.06 to 0.02)
**Cadence (steps·min^-1^)**	111.03 ± 10.96	107.69 ± 11.90	3.34 (0.24 to 6.43)	112.21 ± 11.98	108.68 ± 12.35	3.52 (0.62 to 6.43)	112.79 ± 11.41	109.82 ± 11.90	2.97 (0.00 to 5.94)
**Time (s)**	24.40 ± 3.25	25.55 ± 3.93	-1.15 (-1.96 to -0.341)	48.53 ± 9.05	50.30 ± 9.77	-1.77 (-3.15 to -0.39)	69.33 ± 11.39	72.26 ± 11.75	-2.93 (-5.43 to -0.43)

*Data are expressed as mean ± SD and mean differences (95% confidence intervals) AMP331 – Vicon

† significant differences p < 0.05

**Table 2:** AMP 331 and Vicon gait measures recorded during treadmill walking in healthy young adults

Parameter	2.4 km·h-1			3.2 km·h-1			4.2 km·h-1		

	AMP	Vicon	Mean difference	AMP	Vicon	Mean difference	AMP	Vicon	Mean difference
**Distance (m)**	88.73 ±39.79	117.81 ± 0.68	-29.09† (-51.16 to -7.07)	154.27 ± 12.70	162.77 ± 1.23	-8.50 (-15.66 to -1.58)	188.93 ± 18.85	212.97 ± 1.51	-24.03† (-34.26 to -13.81)
**Step count (steps)**	87.00 ± 40.22	114.13 ± 9.77	-27.13† (-46.57 to -7.70)	139.3 ± 11.7	140.7 ± 11.4	-1.33† (-2.08 to -0.59)	159.33 ± 9.96	160.40 ± 11.13	-1.07 (-2.68 to 0.55)
**Speed (cm·s-1)**	65.60 ± 7.76	65.59 ± 0.1	-0.01 (-4.32 to 4.30)	85.93 ± 6.8	90.16 ± 0.2	-4.07 (-7.85 to -0.02)	104.87 ± 10.06	118.02 ± 0.14	-13.13† (-18.70 to -7.56)
**Step length (cm)**	52.00 ± 7.97	52.04 ± 4.31	-0.05 (-2.97 to 2.88)	55.93± 7.36	57.94 ± 4.79	-2.00 (-4.46 to 0.46)	59.60± 7.50	66.54 ± 4.58	-6.93† (-10.19 to -3.68)
**Cadence (steps·min-1)**	76.57 ± 6.15	76.09 ± 6.51	0.48 (-0.58 to 1.53)	93.12 ± 7.44	93.78 ± 7.59	-0.66† (-1.03 to -0.29)	106.11 ± 6.74	106.93 ± 7.42	-0.83 (-1.96 to 0.31)
**Time (s)**	134.73 ± 59.27	179.59 ± 1.04	-44.86† (-77.51 to -12.20)	179.60 ± 1.92	180.58 ± 1.36	-0.98 (-2.21 to 0.25)	180.20 ± 1.66	180.48 ± 1.29	-0.28 (-1.58 to 1.02)

**Parameter**	**5.8 km·h-1**			**4.2 km·h-1 at 5° incline**		

	**AMP**		**Vicon**	**Mean difference**	**AMP**		**Vicon**	**Mean difference**

**Distance (m)**	251.93 ±22.65		289.23 ± 2.14	-37.49† (-49.84 to -25.14)	178.20 ± 46.53		212.66 ± 1.12	-34.46† (-60.44 to -8.47)
**Step count (steps)**	183.73 ± 9.32		183.73 ± 9.22	0.00 (-0.47 to 0.47)	155.20 ± 10.41		155.40 ± 10.68	-0.20 (-0.63 to 0.23)
**Speed (cm·s-1)**	139.13 ±11.99		160.29 ± 0.11	-20.87† (-27.51 to -14.23)	105.47 ± 8.44		118.36 ± 0.14	-12.67† (-17.32 to -8.01)
**Step length (cm)**	68.87 ± 7.82		78.70 ± 4.00	-9.73† (-13.19 to -6.28)	61.53 ± 6.83		64.14 ± 4.33	-2.47 (-5.34 to 0.40)
**Cadence (steps·min-1)**	121.70 ± 6.22		122.49 ± 6.14	-0.79 (-1.46 to -0.11)	103.27 ± 7.23		103.60 ± 7.12	-0.33 (-0.65 to -0.01)
**Time (s)**	181.20 ± 1.74		180.56 ± 1.34	0.64 (-0.52 to 1.81)	180.33 ± 1.59		180.22 ± 0.95	0.16 (-1.15 to 1.38)

*Data are expressed as mean ± SD and mean difference (95% confidence intervals) AMP331 – Vicon

† significant differences p < 0.05

Statistical analyses were performed using SPSS version 17. Three repeated measures multivariate analysis of variance (RM MANOVAs) were conducted for the over-ground data and five RM MANOVAs were conducted for the treadmill data. Due to the high number of comparisons and the heightened risk of making a Type I error, the alpha level was modified by a Bonferroni adjustment. For the over-ground comparisons, p-values below 0.016 were deemed significant whereas p-values for the treadmill comparisons below 0.01 (α = 0.05) were needed for significance. Mean differences and 95% CI were calculated for studies one and two.

Modified Bland Altman plots (Bland & Altman, 1986), were calculated for the measures of distance, step count and speed for each study. Two-way mixed, single measure intraclass correlation coefficients (ICC) model (3, 1) and 95% confidence intervals were used to assess test re-test reliability for distance and step count in the third study. In addition, the standard error of the measure (SE measure) was calculated in order to express error in the index of measurement and aid clinical interpretation. The formula used was SE measure = standard deviation (SD) x (√1- ICC).

## 3. Results

Good agreement was found between the gait measures recorded by the AMP331 and the Vicon in YA in the over-ground condition (Table 1). On average (combining lap conditions), distance walked differed by 1.8 m, step count by 0.23 steps, speed by 0.2 cm·s^-1^, step length by 1.6 cm, cadence by 3.3 steps·min^-1^, and walking time by 2 seconds. Only gait speed in the 4 lap condition was found to differ significantly (*P* < 0.05) but this difference (0.2 cm·s^-1^) was not considered to be clinically significant.

**Figure 1:**
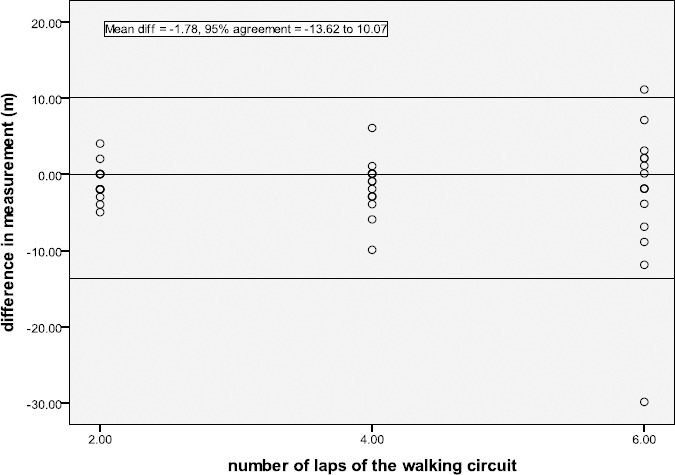
Modified Bland Altman plot for the gait measure of distance (m) during over-ground walking in healthy young adults. Difference in distance measured (AMP331 – Vicon)

Modified Bland Altman plots show the AMP331 generally underestimated distance (Figure 1) and step count, compared to the Vicon. Figure 1 shows the difference in distance measured by the AMP331 and the Vicon for each over-ground walk (2, 4 and 6 laps of the walking circuit). The trend was for the AMP331 to underestimate distance walked at all 3 distances, compared to the Vicon. Combining all walks, the mean distance difference was -1.78m, with 95% of differences falling between -13.62 and 10.07m. The mean step count difference was -0.24, with 95% of differences falling between -1.46 and 0.97. For speed, the mean difference was -0.18 cm·s^-1^, with 95% of the differences falling between -23.78 and 23.41cm·s^-1^.

Forty-three percent of the gait measures recorded by the AMP331 and the Vicon in the treadmill condition were found to be significantly different. The measures to exhibit the greatest error were distance walked, step count and gait speed. In the 2.4 km·h^-1^ treadmill condition, for example, the AMP331 significantly underestimated distance walked by a value of 29.1 m, step count by 27.1 steps and walking time by 45s. Walking at a 5° incline led to an increased underestimation of distance but improved error measurements for the other gait parameters (Table 2).

**Figure 2:**
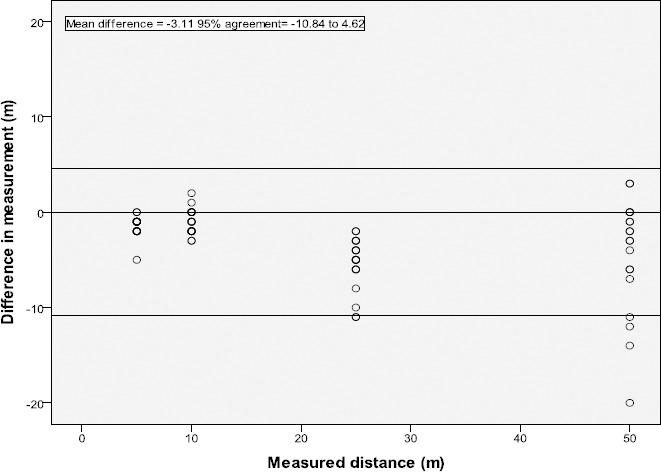
Modified Bland Altman plot for the gait measure of distance (m) during over-ground walking in ICU survivors (walk 2). Difference in distance measured (AMP331 – measured distance)

Nineteen ICU survivors completed two over-ground walks for each distance, with one participant refusing to complete a second walk for each distance. The AMP331 underestimated the mean distance for all distances in both walks, when compared to the measured distance. Results were similar between walks one and two (table 3). The modified Bland Altman plot, of differences between AMP331 distance and measured distance, is shown for walk 2 only (figure 2).

**Table 3:** Difference in gait measures recorded in ICU survivors between the AMP 331 and manually measured parameters

Parameter	Walk 1 Mean (95%CI)	Walk 2 Mean (95%CI)
**Distance (m)**	-2.79 (-3.66 to -1.92)	-3.11 (-3.99 to -2.22)
**Step Count (steps)**	0.93 (0.39 to 1.48)	0.92 (0.44 to 1.40)
**Speed (cm·s^-1^)**	-21.94 (-29.32 to -14.55)	-28.87 (-34.94 to -22.80)

There was a consistent underestimation of distance by the AMP331 at all distances during walks one and two (walk 2 mean difference = -3.11m, 95% agreement of – 10.84 to 4.62m).

There was a similar small underestimation of the mean speed for both walks by the AMP331 compared to stopwatch and direct observation measurement. During walk 2, 95% of all speed differences measured fell between -81.44 and 24.28cm·s^-1^ (mean difference -28.87cm·s^-1^). Contrary to our findings in YA, the AMP331 consistently overestimated the actual step count, with a greater mean difference again in walk 2 (0.92, 95% agreement of -3.27 to 5.11). This overestimation was small, however, and not clinically significant.

In the ICU group, the AMP331 was found to have a high level of reliability for the measure of distance (all distances combined) between walk 1 and walk 2 with an ICC of 0.99 (95% CI 0.98 – 0.99). For walk 2, the SE measure (between AMP331 and observed measure) for distance was 0.78 m. For step count (all distances combined) between walk 1 and walk 2 the ICC was 0.99 (95% CI 0.99 – 1.00). For walk 2, the SE measure for step count was 0.11 steps.

## 4. Discussion

Our results indicate that during over-ground walking at self-selected speed there were no significant differences between the AMP331 and the Vicon when recording spatio-temporal gait parameters. This supports the previous work of Gildenhuys et al 2004, whose subjects completed 5 walks of 200m (Gildenhuys A et al., 2004). Our study has shown the AMP331 to also accurately measure spatio-temporal gait parameters at shorter over-ground walking distances of approximately 30, 60 and 90 metres. There was a trend for the AMP331 to underestimate distance at the shorter walking distances. However, at our largest walking distance there were several occasions where distance was overestimated by the AMP331, although this was not significant overall. These findings support its application in populations who are less active such as those who are debilitated or have chronic diseases.

A study in a paediatric population demonstrated that during continuous walking the AMP331 underestimated walking distance and step count in both healthy children and children with cerebral palsy compared to another accelerometer, the Minimod (Kuo et al., 2009). Consistent with previous findings, during each walk by ICU survivors the AMP331 underestimated distance compared to direct observation. The mean difference (-3.11 m) and 95% limits of agreement (-10.84m – 4.62m) in our study were comparable to results reported in typically developing children measured using the AMP331 (Kuo et al., 2009). Given that participants walked a total of 90 m, an underestimation of 3.11 m is 3% and not considered to be clinically significant.

Recently, the AMP331 has been reported as being more accurate than the Accusplit-AX120 and the MTI-ActiGraph in recording steps/day compared to direct observation in healthy adult American Indians (Pomeroy et al., 2011). Our results highlighted no significant differences between AMP331 and Vicon or direct observation step count. Ninety-five percent of differences in step count between AMP331 and Vicon systems fell between -1.46 and 0.97 steps. The distance walked over-ground by the participants in this study did not affect the accuracy of the AMP331 in recording step count or distance data, as had been reported in previous studies (Karabulut et al., 2005).

We are confident that the AMP331 is as suitable as the Vicon or direct measurement, when measuring distance walked and step count, in our sample of ICU survivors.

The test re-test reliability, for the measures of distance (ICC 0.99 (0.98 – 0.99) walks one and two) and step count (ICC 0.99 (0.99 – 1.00), of individual AMP331 was excellent. This result is important for clinicians who may be using the same AMP331 to monitor changes in PA levels pre and post treatment in a more debilitated population.

The biomechanical differences between over-ground and treadmill walking have previously been reported (Nichols et al., 2000). It has been proposed, that cadence and vertical displacement of the tibia are increased during treadmill walking, leading to an under-estimation of step length. The Stepwatch accelerometer was found to be accurate (99% ± 0.48) and precise in measuring step count at treadmill speeds as slow as 1.6 km/hr compared to manual counts (Foster et al., 2005). A previous study, performed by Karabulut et al(Karabulut et al., 2005), involved participants walking on a treadmill at speeds ranging from 1.6 – 6.4 km/hr. They found the AMP331 underestimated step count at speeds below 4.0km/hr and underestimated distance, by 10%, at speeds greater than 2.4km/hr. When worn for a 24 hour period the AMP331 recorded 18% fewer steps than another ankle mounted pedometer, the SW-3 Ankle. They concluded that ankle mounted accelerometers demonstrated greater accuracy in step count, at slower speeds, than waist mounted and were deemed more suitable in an older population with a slower gait pattern. Results during the treadmill walking component of our study support the findings of Karabulut and colleagues. The AMP331 underestimated step count at each treadmill speed compared to the Vicon, most significantly at the slowest speed. There was no consistent pattern in the measurement error of AMP331 distance. Step length was underestimated at all but the slowest speed. These underestimations increased with increasing treadmill speed. Of importance, such measurement error was not found during over-ground walking where healthy participants were walking at similar speeds. This leads the authors to believe the measurement error of the AMP331, found during the treadmill component of the trial, was due to the effects of treadmill walking on gait pattern, rather than the speed of walking. Other authors have reported issues with the AMP331 rotating around the ankle during walking and not being in line with the Achilles tendon, effecting movement detection (Leenders et al., 2003). We did not observe this to be the case during our data collection.

### 4.1 Study Limitations

It was determined that the over-ground walking track used during Vicon capture needed to be circular due to the Vicon capture range, this limited the researchers’ ability to assess the inter-pod reliability of the AMP331.

## 5. Conclusions

The results from these studies support the use of the AMP331 accelerometer, as a reliable and valid measure of spatio-temporal gait parameters, when used in over-ground walking in healthy adults and ICU survivors. We recommend this system for use in clinical and research environments. Further evaluation is required if the AMP331 is to be used with treadmill walking.
